# Ice in Dysentery

**Published:** 1847-11

**Authors:** 


*Ice in Dysentery.*—One of the editors of the Western Journal of Medicine and Surgery speaks in high terms of the efficacy of ice in dysenteric affections. He states that in an epidemic dysentery prevailing at Louisville during the last summer he "repeatedly witnessed the most pleasing results from this practice, both in children and adults." He continues:—"called to a patient troubled with sick stomach, before giving any medicine, our invariable rule has been to prescribe ice, until the gastric symptoms were relieved, and not a case has yet come under our notice, in which it failed to afford marked and almost immediate relief." We can add our testimony to the safety and advantage of allowing ice in nearly all gastro-intestinal, or other diseases in which it is craved by the patient.

In connection with this subject we would inquire, what effect cold applications to the abdomen externally, exert in dysenteric, and other intestinal affections? We have been informed that they are very generally employed in Boston, Mass., and vicinity. Will some one in that quarter communicate to the profession an answer to the foregoing inquiry ?

